# Network module analysis and molecular docking-based study on the mechanism of astragali radix against non-small cell lung cancer

**DOI:** 10.1186/s12906-023-04148-9

**Published:** 2023-09-28

**Authors:** Wenke Xiao, Yaxin Xu, Jan P. Baak, Jinrong Dai, Lijia Jing, Hongxia Zhu, Yanxiong Gan, Shichao Zheng

**Affiliations:** 1 School of Intelligent Medicine, Chengdu University of Traditional Chinese Medicine, Chengdu, 611137 China; 2 Stavanger University Hospital, Stavanger, 4068 Norway; 3 Dr. Med Jan Baak AS, Tananger, 4056 Norway; 4 School of Pharmacy, Chengdu University of Traditional Chinese Medicine, Chengdu, 611137 China

**Keywords:** Astragali radix, Non-small cell lung cancer, TCGA, Network pharmacology, Molecular docking

## Abstract

**Background:**

Most lung cancer patients worldwide (stage IV non-small cell lung cancer, NSCLC) have a poor survival: 25%-30% patients die < 3 months. Yet, of those surviving > 3 months, 10%-15% patients survive (very) long. Astragali radix (AR) is an effective traditional Chinese medicine widely used for non-small cell lung cancer (NSCLC). However, the pharmacological mechanisms of AR on NSCLC remain to be elucidated.

**Methods:**

Ultra Performance Liquid Chromatography system coupled with Q-Orbitrap HRMS (UPLC-Q-Orbitrap HRMS) was performed for the qualitative analysis of AR components. Then, network module analysis and molecular docking-based approach was conducted to explore underlying mechanisms of AR on NSCLC. The target genes of AR were obtained from four databases including TCMSP (Traditional Chinese Medicine Systems Pharmacology) database, ETCM (The Encyclopedia of TCM) database, HERB (A high-throughput experiment- and reference-guided database of TCM) database and BATMAN-TCM (a Bioinformatics Analysis Tool for Molecular mechanism of TCM) database. NSCLC related genes were screened by GEO (Gene Expression Omnibus) database. The STRING database was used for protein interaction network construction (PIN) of AR-NSCLC shared target genes. The critical PIN were further constructed based on the topological properties of network nodes. Afterwards the hub genes and network modules were analyzed, and enrichment analysis were employed by the R package clusterProfiler. The Autodock Vina was utilized for molecular docking, and the Gromacs was utilized for molecular dynamics simulations Furthermore, the survival analysis was performed based on TCGA (The Cancer Genome Atlas) database.

**Results:**

Seventy-seven AR components absorbed in blood were obtained. The critical network was constructed with 1447 nodes and 28,890 edges. Based on topological analysis, 6 hub target genes and 7 functional modules were gained. were obtained including TP53, SRC, UBC, CTNNB1, EP300, and RELA. After module analysis, Kyoto Encyclopedia of Genes and Genomes (KEGG) pathway analysis showed that AR may exert therapeutic effects on NSCLC by regulating JAK-STAT signaling pathway, PI3K-AKT signaling pathway, ErbB signaling pathway, as well as NFkB signaling pathway. After the intersection calculation of the hub targets and the proteins participated in the above pathways, TP53, SRC, EP300, and RELA were obtained. These proteins had good docking affinity with astragaloside IV. Furthermore, RELA was associated with poor prognosis of NSCLC patients.

**Conclusions:**

This study could provide chemical component information references for further researches. The potential pharmacological mechanisms of AR on NSCLC were elucidated, promoting the clinical application of AR in treating NSCLC. RELA was selected as a promising candidate biomarker affecting the prognosis of NSCLC patients.

**Supplementary Information:**

The online version contains supplementary material available at 10.1186/s12906-023-04148-9.

## Introduction

Lung cancer is the leading cause of cancer-related mortality. It is subdivided as Non-Small-Cell Cancers (NSCLC, 80–85% of all) and Small Cell Lung Cancers (SCLC), as SCLC have a very poor prognosis. The global overall 5-year survival rate of non-small cell lung cancer (NSCLC) is also less than 20% and thus poses a serious threat to public health [1].

NSCLCs are typically divided as operable (patients with TNM stage 1, 2 and 3A) and non-operable (stage 3B and 4, patients with metastatic NSCLC) at the time of diagnosis, whose life expectancy is so poor, that surgery in general is not recommended [2–5]. This leaves systemic platinum-based therapy, radiotherapy, and more recently tyrosine kinase inhibitors target therapy, immune-checkpoint inhibitors, as well as some new frontiers such as Antibody–drug conjugates (ADCs [6]). ADCs are an innovative class of anticancer drugs that combine the strengths of targeted therapy to cytotoxic chemotherapy. Promising results have been demonstrated, but none has been approved for the treatment of NSCLC. The HER2 ADC trastuzumab deruxtecan is the furthest along but other ADCs targeting HER3 and MET will be more valuable as we start to encounter patients with NSCLC harboring EGFR mutations who have developed resistance to the 3rd generation tyrosine kinase inhibitors. ADCs targeting TROP2 holds a particular advantage as TROP2 is known to be expressed in both squamous and nonsquamous NSCLC. NSCLC remains an incurable malignancy resistant to conventional therapy, leading to an overall poor prognosis [7].

Understanding the molecular biological mechanisms of the NSCLC cancer cells have been extensively studied and show geographic/racial variations. For example, in Asian patients, EGFR-mutation status was 51% positive, but in western countries such as Norway, it is much lower (8%). Intercellular signal pathways are also important from the therapeutic point of view. Different signaling pathways mechanisms are known, the EGFR [8], Notch [9], Wnt [10], and Type I IFN signaling pathways [11]. Despite the emerging of new treatment and specific molecular targets, there remains the problem of poor systemic treatment and large toxic and side effects of NSCLC. Developing more efficient therapy to further improve the management of NSCLC is an urgent matter.

Many patients in Asia use Traditional Chinese Medical (TCM) herbs, also because of the serious side effects of western cytotoxic drugs. Indeed, TCM herbs additional to widely used platinum-based chemotherapy, strongly reduces nausea and vomiting [4] and also improve prognosis of stage 3B and 4 NSCLC patients [12]. Over the past decade, the demand for TCM herbs has increased greatly. In 2015, the total production value of the TCM pharma industry in China alone was over 110 billion USD. Due to the significantly increased worldwide interest in the use of TCM herbal medicines, it is expected that the global market value of TCM herbs will significantly increase in the coming years [13, 14].

Astragali radix (AR, Huangqi) is a dominant herb in NSCLC anticancer treatment exhibits immunomodulatory, anti-oxidant, anti-inflammatory and anti-viral properties and shows strong antitumor effects in various tumors including NSCLC [15, 16]. It is speculated that AR mainly exerts its anti-tumor effects by directly inhibiting the proliferation and promoting apoptosis of tumor cells; increasing the efficacy of chemotherapies, potentially preventing tumor cell metastasis and improving TME by enhancing organic or local immunity [16]. McCulloch et al. evaluated the evidence from 34 randomized control trials and found that AR components and AR-based TCM including its combination with platinum-based chemotherapy could reduce the risk of death in 12 months, with improved tumor response data and reduced toxic reaction of chemotherapy [17]. The rapid development of resistance to anticancer agents and their unwanted side effects increase the demand for novel antitumor drugs that can become active against untreatable cancers [18]. Given this background, AR as a popular tonic can play a key role in treatment of NSCLC, because of its effective, safer, and inexpensive than traditional chemotherapeutic drugs. However, the mechanism of AR on NSCLC is unclear. Therefore, it is urgently needed to demonstrate the signaling pathway of AR on NSCLC. As AR exerts therapeutic effects through multi-components, multi-targets and multi-pathways, exploring the mechanisms of TCM against diseases remain a problem due to their complex matrices nature.

Astragaloside IV is one of the main active components from AR, which has attracted much attention in the field of antitumor in recent years [19]. It was found that astragaloside IV can inhibit the proliferation of tumor cells and further inhibit the progression of cancers such as lung cancer [20]. We compared astragaloside IV with Mobocertinib [21], Erlotinib [22], which are marketed for the treatment of NSCLC, and the compound information is shown in Supplementary Table 1.

Network pharmacology, proposed by Li [23], provides a new research paradigm for systematic illumination of TCM mechanism. With the arrival of biological Big Data, bioinformatics had profound implications on the predictive power and reproducibility of results [24]. We have previously shown by detailed network analysis, that LHQW herbal TCM treatment in COVID-19 disease, modulates the inflammatory process, exerts antiviral effects and repairs lung injury. Moreover, it also relieves the “cytokine storm” and improves ACE2-expression-disorder-caused symptoms. These innovative findings give a rational pharmacological basis for and support treating COVID-19 and possibly other diseases with LHQW.

We therefore have applied Network Pharmacology to unravel the working mechanism of Astragali Radix (Huangqi), in Non-Small Cell Lung Cancer. Moreover, Molecular Complex Detection (MCODE [25]) was applied to find densely connected regions of a given network based on topology. Molecular docking refers to the process that a small molecule is spatially docked into a macromolecule and can score the complementary value at the binding sites, which is used for structure‐based drug design [26].

**Fig. 1:**
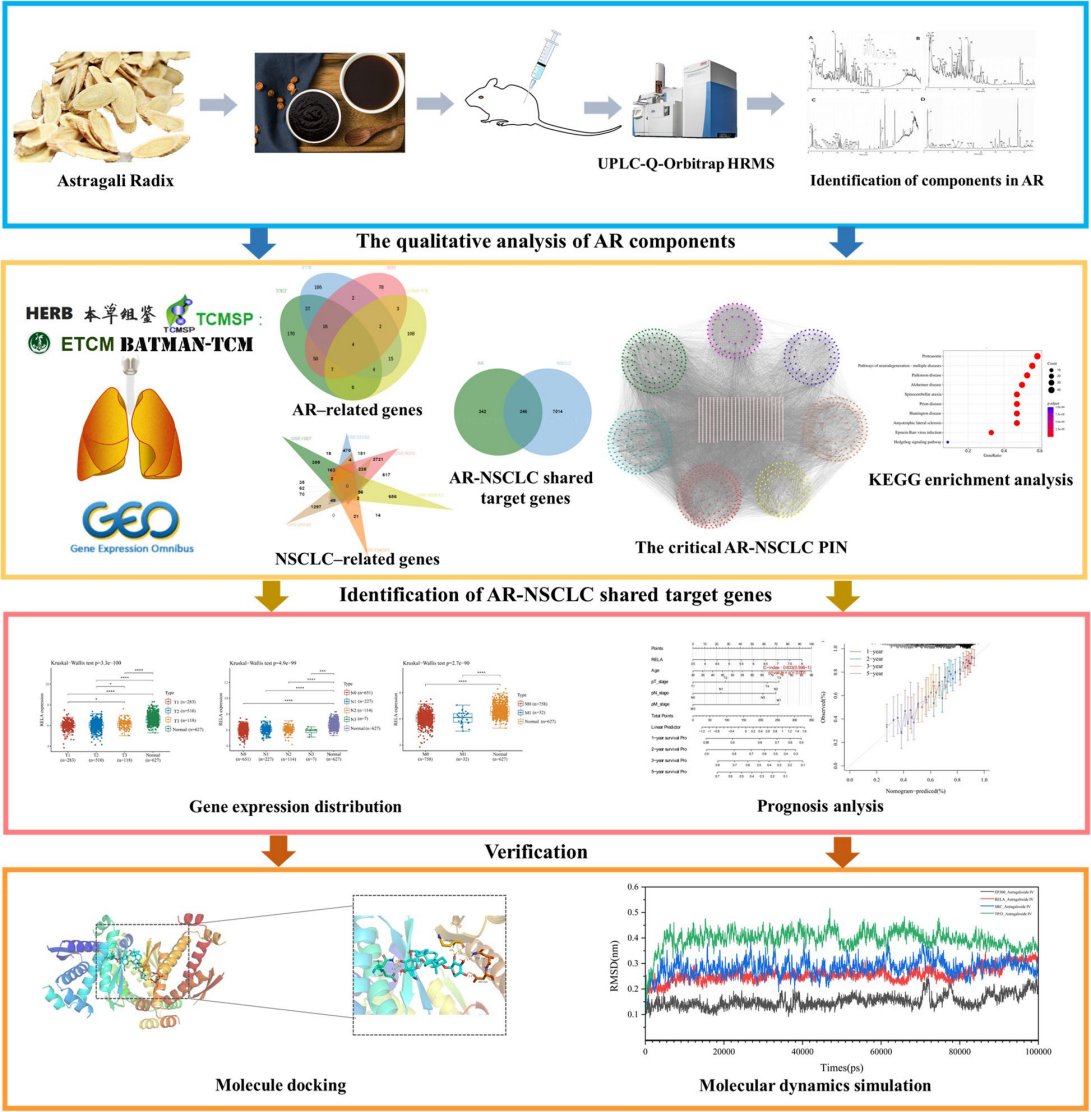
The flowchart of this study was based on UPLC-Q-Orbitrap HRMS, network pharmacology, molecular docking, and molecular dynamics mechanistic simulations to decipher the potential mechanism of astragali radix for the treatment of NSCLC

We firstly employed ultra-high performance liquid chromatography-quadrupole/Orbitrap high resolution mass spectrometry (UPLC-Q-Orbitrap HRMS) to identify the components absorbed in blood of AR. Secondly, we collected information about components, component-related targets, and NSCLC–related genes from extensive databases and identified crucial targets and key pathways based on network module analysis and molecular docking. Finally, the prognostic value of AR-NSCLC hub genes was analyzed using bioinformatics technology. The detailed flowchart of this study is summarized in Fig. 1.

## Materials and methods

### Preparation of samples for analysis

#### Experimental animals

SPF grade male Sprague–Dawley (SD) rats (280 ± 20 g, SCXK, Jing, 2019–0010) were purchased from SPF (Beijing) Biotechnology Co., Ltd. (Beijing, China). All rats were housed in the specific-pathogen-free grade animal observation room of Chengdu University of Traditional Chinese Medicine (SYXK, Chuan, 2020–124), which was set at 21 ± 1℃ and 60% relative humidity with a 12 h light/dark cycle. Rats were deprived of food for 12 h before the start of experiment, but permitted of free access to drinking water. This study was performed in accordance with the guidelines of the National Health Institutes of China and approved by the Animal Ethics Committee of Chengdu University of Traditional Chinese Medicine (Chengdu, China, Ethical approval number: 2018–15).

#### Preparation of plasma samples

Astragali radix was obtained from affiliated hospital of Chengdu University of Traditional Chinese Medicine. Briefly, astragali radix was soaked in 8-fold mass of purified water for 30 min and then decocted for 50 min twice. After being filtered with gauze, the two filtrates were combined and concentrated to 1 g/mL using a rotary evaporator at 40 °C for intragastric administration.

After 7 days of acclimation, SD rats were randomly distributed in two groups including control and AR group (2.7 ml/100 g), nine rats in each group. The control group was given equal volume distilled water.

Blood was collected at 30,60 and 120 min after administration, and then the rats were anesthetized with pentobarbital sodium. Blood samples of 3 rats were collected each time, then the rats were dragged by the neck to death after the experiment. The collected blood was centrifuged for 15 min at 4000 r/min, 4 ℃. 250 µL plasma was mixed with 1000 µL acetonitrile (chromatographically pure) and performed vortex for 4 min, then centrifuged for 15 min at 13,000 r/min, 4 ℃. The supernatant was transferred to another clean Eppendorf tube, and evaporated until dry using a mild nitrogen gas stream at 25 ℃. Add 150 µL 0.01% hydrochloric acid methanol (1:1) mixed solution for redissolution, centrifuge at 13,000 r/min, 4 ℃ for 15 min, and suck the clear solution into the tube. The plasma test samples collected at three time points in the same administration group were mixed and filtered through a 0.22 µm filter for UPLC-Q-Orbitrap HRMS analysis.

#### Preparation of AR samples for analysis

20 ml of AR decoction was weighed accurately, added with 100 ml of 75% methanol solution, and weighed accurately again. After refluxing for 60 min, 75% methanol solution was used to make up the lost weight and then filtered. 90 ml of filtrate was evaporated using a rotary evaporator at 40 °C. The dried extracts were redissolved in methanol to 10 mL. The redissolved methanolic solution was filtered with a 0.22 μm filter before injecting into the UPLC-Q-Orbitrap HRMS system.

### UPLC-Q-Orbitrap HRMS analysis

The qualitative analysis of chemical components was performed using Ultra Performance Liquid Chromatography (UPLC) system coupled with Q-Orbitrap HRMS/MS (Thermo Fisher Scientific, United States). The sample was separated by using Thermo Scientific Accucore^TM^C18 (3 mm × 100 mm, 2.6 µm) eluted with a mixture of 0.1% formic acid (A) and acetonitrile (B). The proportion of acetonitrile (B) was increased from 1–20% (0–8 min), 20–30% (8–10 min), 30–35% (10–12 min), 35–40% (12–14 min), 40–55% (14–23 min), 55–70% (23–27 min), 70–80% (27–30 min), 80–99% (30–37 min), 99% (37–38 min). The flow rate was 1 mL min − 1, and the column temperature was set at 40 °C. The injection volume was 5 μL.

Mass detection was performed on a Q-Exactive Orbitrap Mass Spectrometer equipped with an Electron Spray Ionization source in positive and negative ion mode. Mass conditions were as follows: the spray voltage in positive/negative mode was 5.0 kV/3.0 kV; sheath gas (N2) flow rate of 50 L/h Pa; and the probe heating temperature is 350 ℃. In the automatic MSE mode, the CLT-GND voltage (+) and CLT-GND voltage (-) are 20 V and 15.9 V respectively. The full-scan mass spectrum was recorded in m/z 50–2,035 Da. All data were acquired and processed by Compound Discoverer 2.9 software (Thermo Fisher Scientific, FL, United States).

### Obtaining the AR targets and NSCLC related genes

Four database were used to search AR’s components targets: TCMSP (Traditional Chinese Medicine Systems Pharmacology) database (https://tcmspw.com/) [27], ETCM (The Encyclopedia of TCM) database (http://www.tcmip.cn/ETCM/index.php/Home/Index/) [28], HERB [29] (A high-throughput experiment- and reference-guided database of TCM) database (http://herb.ac.cn/) and BATMAN-TCM [30] (a Bioinformatics Analysis Tool for Molecular mechanism of TCM) database (http://bionet.ncpsb.org.cn/batman-tcm/). A union of the search results was used to establish an AR related target genes.

NSCLC related genes were the union of differentially expressed genes (DEGs) obtained from the gene expression profiles including GSE1987 [31], GSE33532 [32], GSE7670 [33], GSE103512 [34], GSE134381 [35] and GSE29249 [36] in GEO (Gene Expression Omnibus) database. We firstly downloaded the raw data as MINiML files. And then we normalized the probe expression profile by the preprocessCore package in R software (version 3.4.1). Next, probes were converted to gene symbols according to the platform annotation information of the normalized data. Probes with more than one gene were eliminated using the removeBatchEffect function of limma R package. When multiple probes can be converted to the same gene, the probe with the average expression level is selected to represent that gene. For each gene expression profiles, we performed the differential expression analysis by using the R package Limma 3.42.2. In brief, we firstly fitted linear models from the normalized gene expression matrix by using the ‘lmFit’ function, and then computed the empirical Bayes statistics by using the ‘eBayes’ function. Finally, we selected the genes with sufficient expression difference. For example, the parameters of adjusted *p* value < 0.05 and logFC cutoff criteria ≥ 0.5 are upregulated and adjusted *p* value < 0.05 and logFC cutoff criteria ≤ -0.5 are downregulated were selected [37]. The AR-NSCLC shared genes were obtained by intersecting the AR target genes and the NSCLC related genes.

### Constructing component-target network, protein–protein interaction network (PIN) and critical PIN

Based on the AR-NSCLC shared target genes, a component-target network was constructed using Cytoscape version 3.9.0 [38]. Then, we imported AR-NSCLC shared target genes into the STRING database (http://string-db.org/) to obtained the protein–protein interactions (PPI) used for constructing the AR-NSCLC PIN. The PPI confident score was set at 0.9 to ensure the credibility of the results. Meanwhile, we performed the network analysis by means of Analyze Network plugin in Cytoscape to calculate the betweennesscentrality, closenesscentrality and degree of each node in AR-NSCLC network [39]. Finally, we filtered genes according to the score of betweennesscentrality, closenesscentrality and degree higher than the median value. We used the filtered genes to construct the primary critical PIN based on AR-NSCLC PIN background. The filter process was conducted again to acquire the final critical AR-NSCLC PIN.

### Analysis of network topological properties

Topological properties have become very popular to gain an insight into the organization and the structure of the complex networks [40]. Therefore, the topological parameters such as degree distribution and average shortest path were analyzed by Network Analyzer in Cytoscape [40]. Properties of scale-free and small word of the critical AR-NSCLC network were also investigated to assess the biological feasibility.

### Hub genes, module and enrichment analysis of the critical AR-NSCLC PIN

CytoHubba plugin in Cytoscape was employed to calculate the degree, closeness and betweenness of nodes in critical AR-NSCLC PIN. We select the nodes as the hub genes whose score of degree, closeness and betweenness was in the top 10. Module analysis was performed by the MCODE plugin in Cytoscape to explore the significant modules in critical AR-NSCLC PIN. The advanced options set as degree cutoff = 2, K-Core = 2, and Node Score Cutoff = 0.2. Enrichment analysis, including GO (gene ontology) and KEGG (Kyoto Encyclopedia of Genes and Genomes) pathway analysis, was performed for the genes in modules to reveal the underlying mechanism through biological processes, cellular components, molecular function and key signaling pathways. ClusterProfiler package (version: 3.18.0) in R was employed to analyze enrichment analysis. The box plot was implemented by the R software package ggplot2; PCA graphs were drawn by R software package ggord; the heat map is displayed by the R software package pheatmap. All the above analysis methods and R package were implemented by R foundation for statistical computing (2020) version 4.0.3.

### Molecular docking

The molecular docking was performed in Autodock Vina software to evaluate the accuracy of network analysis results. We downloaded the 2D structure for the molecule ligands from the PubChem database (https://pubchem.ncbi.nlm.nih.gov/). The 3D structure of protein was downloaded in RCSB PDB database (https://www.rcsb.org/). ChemBio 3D software was used to calculate and export the minimized energy stricter. Then the protein receptors were performed to remove water molecules, separate proteins, add nonpolar hydrogens, calculate Gasteiger charges of the structures in AutoDock Tools 1.5.6, and saved as PDBQT files. PyMOL 2.3.0 software was used to set the active pocket sites where molecule ligands bind and visualize the docking results. In addition, analyze all hydrogen bonding and hydrophobic interactions with ProteinPlus [41].

### Molecular dynamics simulation

Gromacs 2022.3 [42] (Van Der Spoel et al., 2005) was used to analyze the molecular dynamics (MD) simulation to check the stability of the protein–ligand complexes. We imported the structure files of proteins and ligand into Gromacs separately, and generated topology files and simulation boxes by using the pdb2gmx and gmx editconf commands. The structures of proteins and ligand were minimized and simulated using the gmx grompp and gmx mdrun commands. The molecular dynamics simulation system was first energy minimization using the steepest descent method, followed by 100,000 steps of isothermal isovolumetric (NVT) and isothermal isobaric (NPT) equilibria with coupling constants of 0.1 ps and durations of 100 ps. Finally, a free-fractional dynamics simulation was run, with a step size of 2 fs. Molecular dynamics simulations of proteins and ligand were performed for up to 100 ns using the gmx grompp and gmx mdrun commands, and conformational information during the simulations was recorded. The root mean square deviation (RMSD) of each complex was analyzed to measure the stability of the complex system according to the degree of the molecular structure change. The root mean square fluctuation (RMSF) of the identified complexes was analyzed to understand the relative fluctuation of proteins. The receptor-ligand binding free energy was calculated using the Molecular Mechanics Poisson-Boltzmann Surface Area (MMPBSA) method in the 30 ns MD simulation trajectory.

### Prognostic value of hub genes

To see whether hub genes were related to prognostic significance, survival analysis was performed in the R environment using TCGAbiolinks [43]. The gene expression quantification data and clinical information of NSCLC patients were downloaded from The Cancer Genome Atlas (TCGA) database (https://portal.gdc.cancer.gov/) which has generated comprehensive, multi-dimensional maps of the key genomic changes in various types of cancers. RNA-seq data (level 3) including 1017 NSCLC tissues and 108 normal tissues, and corresponding clinical information of NSCLC were obtained.

Univariate and multivariate Cox regression analyses were performed to identify prognostic factors for NSCLC. The forest was used to show the *P* value, HR and 95% CI of each variable through ‘forestplot’ R package. Thereafter, nomogram was developed based on the results of multivariate cox proportional hazards analysis to predict the 5-year overall recurrence. The nomogram provided a graphical representation of the factors which could be used to calculate the risk of recurrence for an individual patient by the points associated with each risk factor through ‘rms’ R package. The predictive accuracy of the nomogram was assessed by calibration plot [44]. Null hypotheses of no difference were rejected if *p*-values were less than 0.05.

## Results

### Components analysis by UPLC-Q-Orbitrap HRMS

**Fig. 2:**
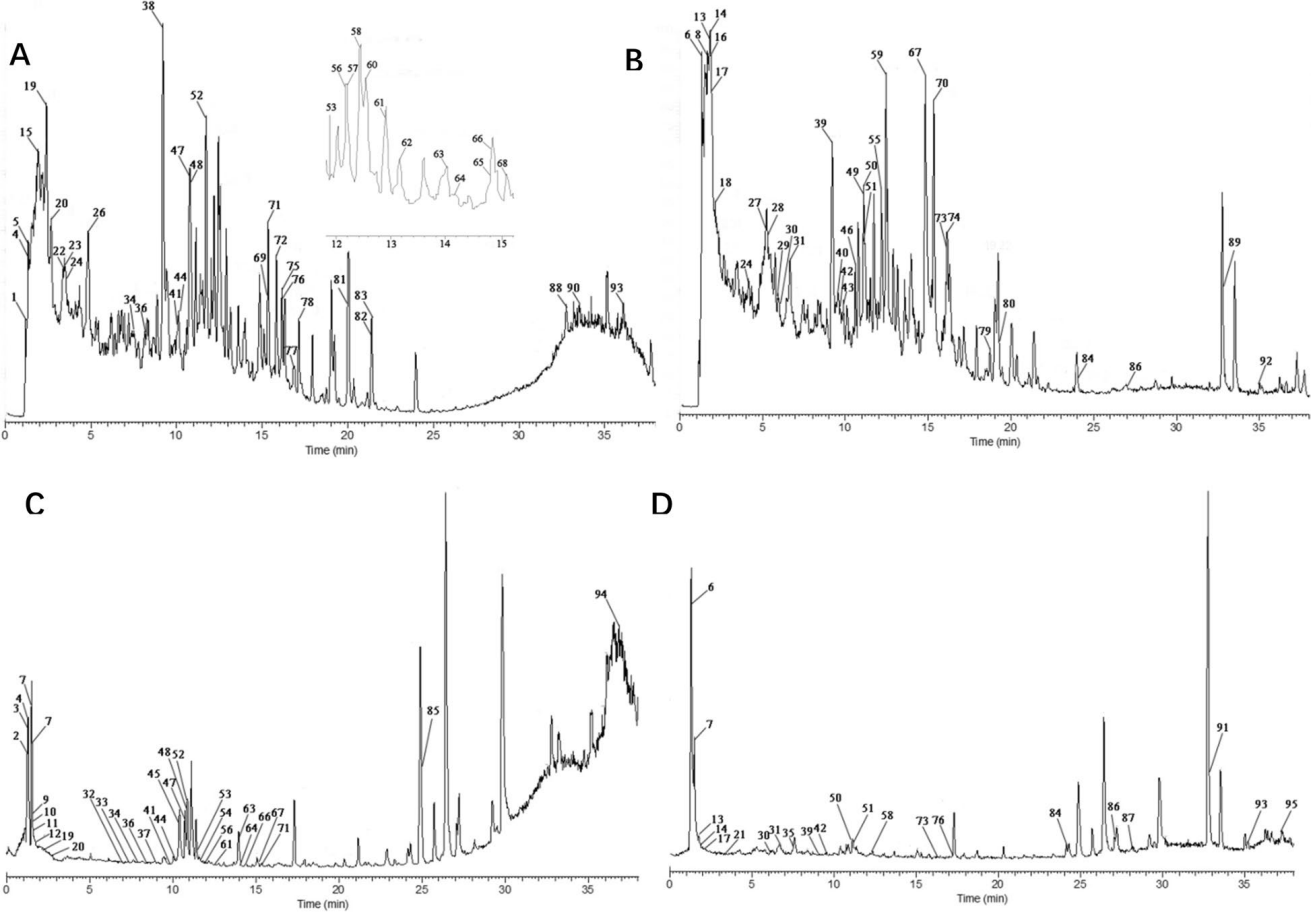
Representative UPLC-Q-Orbitrap HRMS total ion chromatograph of AR in positive mode (**A**) and negative mode (**B**), and AR absorbed in blood in positive mode (**C**) and negative mode (**D**)

The total ion chromatograms of AR and AR absorbed in blood in the positive and negative ion mode is shown in Fig. 2. The tentative identification of components was conducted by comparing retention times to external standards and MS data. Compared to blank plasma data, 77 component peaks were observed from AR plasma. Among the migration components in AR plasma, 35 components were from AR, as shown in Table 1.

**Table 1:** Identification of components in AR and AR absorbed in blood by UPLC-Q-Orbitrap HRMS

NO	RT (min)	Adducts	Observed(m/z)	Error(ppm)	MS fragment	Formula	Component
1	1.18	[M + H]	146.1055	-0.18	147.1128, 130.0864, 123.9652, 102.0918, 93.0371, 84.0814	C_6_H_14_N_2_O_2_	DL-Lysine
2	1.31	[M + H]	176.0909	-0.24	177.0985, 160.0719, 149.0299, 120.0659, 118.0503, 102.0555	C_5_H_12_N_4_O_3_	Canavanine
3	1.33	[M + H]	137.0479	1.54	138.0552, 136.0397, 110.0606, 96.045, 94.0658	C_7_H_7_NO_2_	Trigonelline
4	1.34	[M + FA-H]	342.1161	-0.22	341.1087, 229.315, 179.0557, 161.0449, 149.0449	C_12_H_22_O_11_	Sucrose
5	1.47	[M-H]	162.0521	-4.61	161.0449, 143.0334, 131.034, 101.0235, 99.0442	C_6_H_10_O_5_	3-Hydroxy-3-methylglutaric acid
6	1.78	[M-H-H_2_O]	324.0359	0.03	308.63, 211.0009, 202.919, 111.0191, 96.9686, 78.958	C_9_H_13_N_2_O_9_P	Uridine monophosphate (UMP)
7	1.81	[M-H]	192.0267	-2.60	191.0192, 173.0084, 154.9981, 147.0298, 129.0185, 111.0078	C_6_H_8_O_7_	Citric acid
8	1.92	[M + H]	329.0533	2.22	330.0603, 273.1199, 213.016, 202.0725, 196.0732, 136.0621	C_10_H_12_N_5_O_6_P	Adenosine3′5'-cyclic monophosphate
9	1.95	[M-H]	345.0478	1.12	344.0401, 192.9904, 150.0414, 133.0147, 126.03	C_10_H_12_N_5_O_7_P	Guanosine cyclic monophosphate
10	1.97	[M-H]	244.0695	-0.14	243.0622, 200.0559, 182.0459, 168.7182, 140.0346, 122.0238	C_9_H_12_N_2_O_6_	Uridine
11	2.01	[M-H]	181.0739	-0.20	182.0797, 165.0548, 147.0442, 136.0758, 123.0443	C_9_H_11_NO_3_	2-Hydroxyphenylalanine
12	2.40	[M + H]	267.0979	4.45	268.1044, 196.4948, 152.0552, 137.0459, 136.0621	C_10_H_13_N_5_O_4_	Adenosine
13	2.68	[M + H]	283.0923	2.34	284.098, 247.9412, 198.3079, 154.025, 152.057	C_10_H_13_N_5_O_5_	Guanosine
14	3.36	[M + H]	281.1133	3.07	282.12, 253.4694, 219.9867, 147.0652, 136.062	C_11_H_15_N_5_O_4_	2'-O-Methyladenosine
15	3.52	[M + H]	151.0501	4.34	152.057, 135.0304, 128.0457, 124.04, 110.0354	C_5_H_5_N_5_O	Guanine
16	3.53	[M + H]	165.0788	-1.39	166.0863, 148.039, 138.0546, 131.0493, 120.081	C_9_H_11_NO_2_	L-Phenylalanine
17	4.13	[M-H]	256.0584	0.30	255.0508, 246.9623, 211.0602, 193.0503, 179.0343, 165.055	C_11_H_12_O_7_	Piscidic acid
18	4.85	[M + H]	145.0530	1.80	146.0605, 140.3426, 128.071, 118.0656, 110.0605	C_9_H_7_NO	4-Indolecarbaldehyde
19	5.19	[M-H]	462.1377	0.66	461.1295, 443.1768, 404.2693, 281.067, 239.0551, 209.0454	C_19_H_26_O_13_	Sibiricose A3
20	5.39	[M-H]	159.0889	-4.26	158.0815, 133.1278, 116.0708, 114.0915, 112.0508	C_7_H_13_NO_3_	N-Acetylvaline
21	5.94	[M-H]	176.0679	-3.08	175.0606, 157.05, 131.0705, 129.055, 115.0392	C_7_H_12_O_5_	2-Isopropylmalic acid
22	6.16	[M-H]	168.0418	-2.96	167.0341, 152.0107, 139.0027, 123.0443, 108.0207	C_8_H_8_O_4_	Vanillic acid
23	6.64	[M-H]	160.073	-4.09	159.0656, 141.055, 130.9827, 115.0756, 97.0649	C_7_H_12_O_4_	Pimelic acid
24	7.38	[M + H]	376.1399	4.24	377.1461, 359.1355, 316.13, 293.1228, 285.0993, 277.6668	C_17_H_20_N_4_O_6_	Riboflavin
25	8.13	[M + H]	152.0479	3.75	153.055, 135.0809, 125.0601, 111.0446, 110.0368	C_8_H_8_O_3_	Vanillin
26	9.19	[M + H]	446.1210	-0.70	447.1296, 285.0761, 270.0526, 253.0499, 269.0447	C_22_H_22_O_10_	Glycitin
27	9.21	[M-H]	283.0612	-0.02	283.0611, 068.0377, 251.0346, 239.0348, 224.0475	C_16_H_12_O_5_	Calycosin
28	9.56	[M-H]	246.1004	-0.23	245.093, 203.0821, 201.1025, 186.0555, 183.0924, 116.0496	C_13_H_14_N_2_O_3_	N-Acetyl-DL-tryptophan
29	9.73	[M + H]	432.1069	2.96	433.23, 354.3557, 332.1812, 299.1601, 271.0605	C_21_H_20_O_10_	Genistin
30	9.99	[M-H]	610.1536	0.32	609.1458, 563.1987, 433.0979, 415.0896, 371.1057, 193.0501	C_27_H_30_O_16_	Rutin
31	10.05	[M-H]	460.1009	0.72	459.093, 283.061, 268.0376, 239.0345, 224.0483	C_22_H_20_O_11_	Wogonoside
32	10.07	[M + H]	284.0687	0.95	285.0761, 270.0522, 263.1551, 253.0495, 225.0552	C_16_H_12_O_5_	Biochanin A
33	10.59	[M-H]	262.1418	0.97	261.1343, 187.097, 169.0862, 161.3586, 143.1068	C_12_H_22_O_6_	9-(2,3-dihydroxypropoxy)-9-oxononanoic acid
34	10.72	[M + H]	369.1578	0.46	370.1654, 352.1547, 339.1221, 336.1238, 306.0869, 290.094	C_21_H_23_NO_5_	Allocryptopine
35	10.80	[M + H]	488.1319	-0.01	489.1417, 471.3192, 453.3059, 371.2288, 296.1868	C_24_H_24_O_11_	6''-O-Acetylglycitin 6''-O-Acetyl Daidzein
36	11.09	[M-H]	188.1043	-3.18	187.097, 169.0868, 143.1068, 123.0718, 125.0963	C_9_H_16_O_4_	Azelaic acid
37	11.11	[M-H]	166.0624	-3.63	165.055, 147.0443, 135.0453.121.0649, 119.0493	C_9_H_10_O_3_	3-Phenyllactic acid
38	11.18	[M-H]	270.0529	0.21	269.0454, 241.0502, 237.0554, 225.0553, 231.0549	C_15_H_10_O_5_	Genistein
39	11.72	[M + H]	430.1266	0.44	431.1339, 299.0935, 269.0812, 254.0577, 237.0549	C_22_H_22_O_9_	Ononin
40	11.91	[M + H]	254.0588	3.36	255.0656, 237.0548, 227.0707, 219.1373, 209.0607	C_15_H_10_O_4_	Daidzein
41	12.13	[M + H]	256.0738	0.89	257.0812, 242.0579, 239.0706, 223.4929, 215.0699, 147.0444	C_15_H_12_O_4_	Isoliquiritigenin
42	12.17	[M-H]	515.2923	1.24	514.2841, 124.0064, 106.98, 79.9564, 66.3379	C_26_H_45_NO_7_S	Taurocholic acid
43	12.19	[M + H]	462.1529	0.65	463.2562, 327.1236, 301.1072, 286.0807, 241.086, 167.0706	C_23_H_26_O_10_	Methylnissolin-3-O-glucoside
44	12.21	[M + H]	187.0629	-2.47	188.071, 181.1584, 170.0604, 167.1744, 146.0603	C_11_H_9_NO_2_	Indole-3-acrylic acid
45	12.41	[M + FA-H]	464.1683	0.00	463.1581, 443.6863, 399.105, 341.4269, 310.5716, 301.108	C_23_H_28_O_10_	Isomucronulatol 7-O-glucoside
46	12.44	[M-H]	202.1201	-2.15	201.1127, 194.9919, 183.1021, 157.1229, 139.112	C_10_H_18_O_4_	3-tert-Butyladipic acid
47	12.54	[M + H]	516.1269	0.22	517.1362, 419.5182, 307.0505, 269.0813, 254.0581	C_25_H_24_O_12_	Formononetin 7-O-glucoside-6''-O-malonate
48	12.88	[M + H]	270.0892	-0.07	271.097, 253.1623, 239.0707, 161.06, 147.0442, 137.06	C_16_H_14_O_4_	Medicarpin
49	13.96	[M + H]	284.0684	-0.14	285.0762, 270.0527, 257.0817, 253.0500, 249.7172	C_16_H_12_O_5_	Glycitein
50	14.14	[M + H]	300.0639	1.62	301.0711, 286.0476, 273.0763, 269.0448, 258.0525	C_16_H_12_O_6_	Diosmetin
51	14.75	[M + H]	194.1309	1.07	195.1385, 177.1278, 107.0861, 95.0862, 93.0705, 81.0706	C_12_H_18_O_2_	Sedanolide
52	14.80	[M + H]	164.1203	1.24	165.1277, 147.1171, 137.096, 123.1171, 121.1016, 119.086	C_11_H_16_O	Jasmone
53	14.85	[M-H]	330.2406	-0.66	329.2332, 311.2210, 293.2120, 228.8605, 229.1444	C_18_H_34_O_5_	(15Z)-9,12,13-Trihydroxy-15-octadecenoic acid
54	15.11	[M-H-H_2_O]	784.4620	1.34	782.57, 758.5696, 703.5757, 552.4032, 414.3221	C_41_H_68_O_14_	Astragaloside IV
55	15.27	[M + H]	315.2418	2.69	316.2485, 257.1745, 175.4363, 155.1436, 149.3798, 95.0861	C_17_H_33_NO_4_	Decanoylcarnitine
56	15.34	[M-H]	408.2877	0.23	407.2801, 389.2693, 371.26, 363.2912, 353.2492	C_24_H_40_O_5_	Cholic acid
57	15.34	[M + H]	268.0738	1.05	269.0813, 254.0578, 237.0551, 226.0629, 231.0914	C_16_H_12_O_4_	Formononetin
58	15.85	[M + H]	298.0853	4.10	299.0919, 284.0683, 266.0578, 255.0656, 238.0628	C_17_H_14_O_5_	Mosloflavone
59	16.10	[M-H]	328.2249	-0.12	327.2118, 309.2084, 291.1954, 239.1292, 229.1442, 211.1335	C_18_H_32_O_5_	Corchorifatty acid F
60	16.14	[M-H]	449.3142	0.08	448.3066, 386.3057, 324.6598, 204.116, 192.1167, 88.6858	C_26_H_43_NO_5_	Glycoursodeoxycholic acid
61	16.15	[M + H]	272.1779	1.05	273.1854, 255.1743, 245.1904, 237.1637, 227.1791	C_18_H_24_O_2_	19-Norandrostenedione
62	16.20	[M-H]	499.2972	0.93	498.2891, 342.0472, 152.6066, 124.0066, 106.9798	C_26_H_45_NO_6_S	Taurochenodeoxycholic acid
63	16.88	[M + H]	942.5195	0.71	943.5283, 599.394, 581.386, 411.3629, 141.0184, 85.0291	C_48_H_78_O_18_	Soyasaponin I
64	17.15	[M + H]	454.3450	0.64	455.3525, 437.3423, 419.3319, 297.222, 279.2116	C_30_H_46_O_3_	Dehydrotrametenolic acid
65	18.71	[M-H]	294.1832	0.35	293.1757, 236.1051, 221.1543, 220.1465, 205.1231	C_17_H_26_O_4_	6-Gingerol6-Gingerol
66	19.32	[M-H]	392.2929	0.67	391.2855, 378.2416, 253.8428, 197.8076, 160.8415	C_24_H_40_O_4_	Deoxycholic acid
67	19.96	[M + H]	512.3489	-2.42	513.3558, 432.4901, 320.8299, 269.7129, 241.5211	C_32_H_48_O_5_	3-Acetyl-11-keto-β-boswellic acid
68	20.35	[M + H]	182.0735	1.56	183.0806, 159.9691, 154.9904, 150.9572, 141.9834, 105.034	C_13_H_10_O	Benzophenone
69	21.38	[M + H]	299.2829	1.63	282.2798, 264.2694, 252.2685, 121.1015, 109.1018	C_18_H_37_NO_2_	D-Sphingosine
70	24.13	[M-H]	294.2197	0.83	293.2119, 275.2017, 235.1701, 231.2118, 221.1547	C_18_H_30_O_3_	13(S)-HOTrE
71	27.07	[M-H]	489.3589	-0.82	489.3589, 471.3436, 413.341, 326.8288, 280.116	C_30_H_50_O_5_	Cycloastragenol
72	32.72	[M + H]	255.2564	0.89	256.264, 185.6238, 176.7254, 148.2043, 144.1391	C_16_H_33_NO	Hexadecanamide
73	32.84	[M-H]	304.2401	-0.58	303.2327, 265.3597, 259.2428, 205.1955, 122.1624	C_20_H_32_O_2_	Arachidonic acid
74	33.26	[M + H]	281.2721	0.96	282.2797, 265.2531, 247.2423, 226.2181, 212.2006	C_18_H_35_NO	Oleamide
75	35.15	[M-H]	256.2401	-0.47	255.2329, 220.258, 119.0493	C_16_H_32_O_2_	Palmitic acid
76	36.08	[M + H]	283.2881	2.17	284.2953, 246.3729, 197.5283, 189.7503, 128.1083	C_18_H_37_NO	Stearamide
77	37.69	[M-H]	456.3604	0.00	455.3531, 437.3395, 409.3479, 353.2882, 283.2651	C_30_H_48_O_3_	Ursolic acid

### Identification of AR-NSCLC shared target genes

**Fig. 3:**
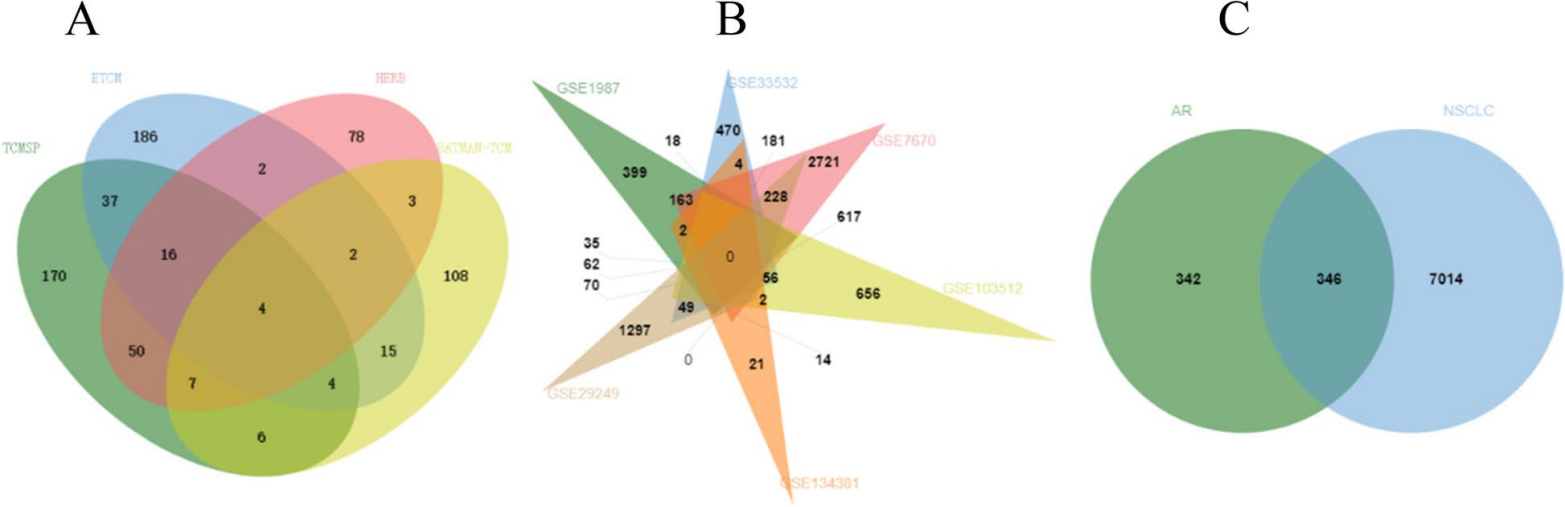
Identification of the AR-NSCLC shared target genes. **A** The AR–related genes by taking a union of all the results from 4 databases. **B** The NSCLC–related genes by taking a union of all the results from 7 gene expression profiles. **C** The AR-NSCLC shared target genes by taking an intersection of AR target genes and NSCLC–related genes

An overall 688 AR components target genes were gained after removing duplication (Fig. 3A). 294, 266, 162 and 149 AR components target genes were obtained from TCMSP, ETCM, HERB and BATMAN-TAM database, respectively. Besides, 784, 918, 4193, 1667, 59 and 1859 NSCLC-related genes were obtained from GSE1987, GSE33532, GSE7670, GSE103512, GSE134381 and GSE29249, respectively. After combining multiple gene expression profiles results and removing duplication, a total of 7360 NSCLC- related genes were acquired (Fig. 3B). Further, through taking an intersection of the AR-target genes and NSCLC-related genes, we finally gained the AR-NSCLC shared target genes (Fig. 3C).

### Component-target network and critical AR-NSCLC network

**Fig. 4:**
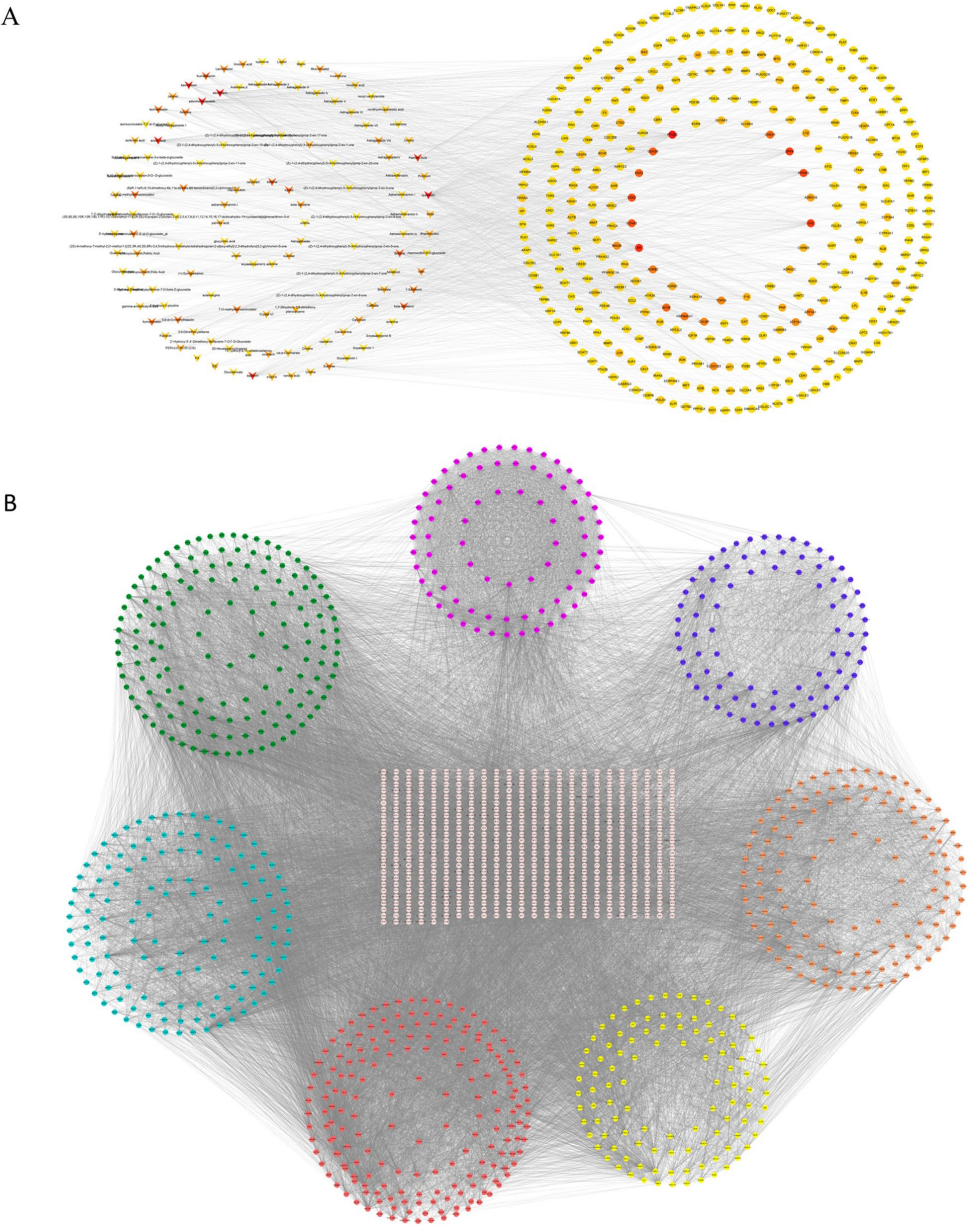
Protein–protein interaction network. **A** The component-targets interaction network. Triangles represent the components in AR. Circles represent the AR-NSCLC shared targets. The larger the degree of the node, the darker the color of the node. **B** The critical AR-NSCLC PIN and eight significant modules identified from the critical AR-NSCLC PIN via MCODE with a score of > 5. The circle represents module1, module 2, module 3, module 4, module 5, module 6 and module 7 respectively, starting from the red in a clockwise direction

The component-target network was visualized by Cytoscape with 467 nodes and 1244 edges (Fig. 4A). Among the 346 genes, PTGS2 is the most targeted gene by AR components. Based on the AR-NSCLC shared target genes, protein–protein interactions derived from STRING database were imported into Cytoscape to visualize the AR-NSCLC network with 4313 nodes and 62,617 edges. After two filtrations of topological properties including betweennesscentrality, closenesscentrality and degree, the critical AR-NSCLC network was constructed with 1447 nodes and 28,890 edges (Fig. 4B).

### Topological analysis

All the topological parameters of AR-NSCLC PIN and critical AR-NSCLC PIN were calculated and shown in Table 2.

**Table 2:** The simple parameters of protein interaction network

Parameters	AR-NSCLC PIN	critical AR-NSCLC PIN
Number of nodes	4313	1447
Clustering coefficient	0.444	0.417
Network diameter (radius)	9 (5)	5 (3)
Network centralization	0.071	0.143
Shortest paths	18,597,656	2,092,362
characteristic path length	3.380	2.608
Network heterogeneity	1.131	0.807

The network diameter is the longest distance between any pair of vertices and the radius of a graph is the minimum eccentricity of any vertex. Network centralization is a network index that measures the degree of dispersion of all node centrality scores in a network. And network heterogeneity quantifies the degree of uneven distribution of the network

**Fig. 5:**
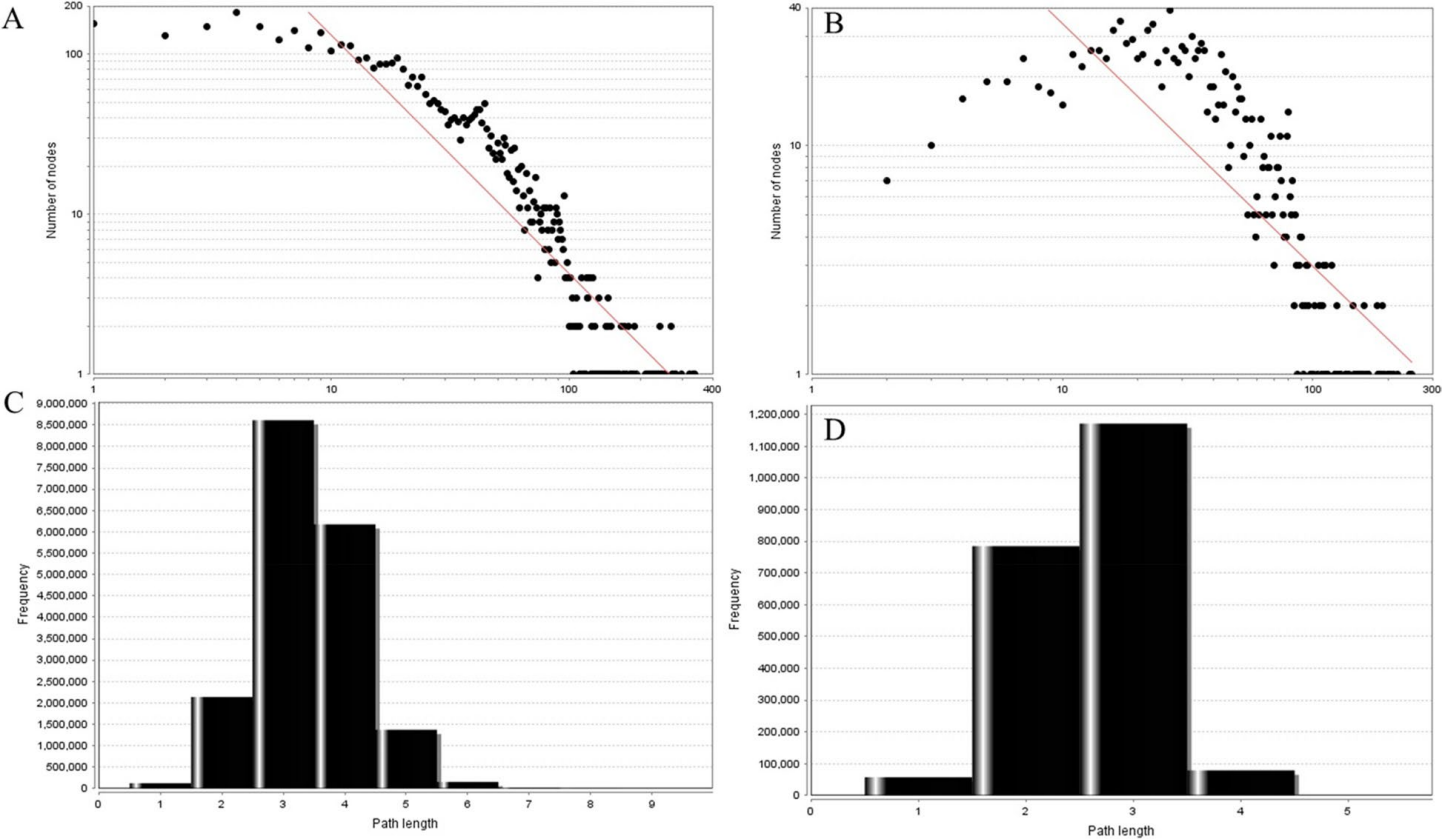
Topological properties of the network. **A** The degree distribution of AR-NSCLC PIN; **B** The degree distribution of critical AR-NSCLC PIN; **C** The shortest path length distribution of AR-NSCLC PIN; **D** The shortest path length distribution of critical AR-NSCLC PIN

Biological networks have been proposed to have scale-free topology whose degree distribution follows a power law distribution *P(k)* ~ *k *^*−γ*^ (*γ* < 3) [45]. As shown in Fig. 5(A and B), the degree distribution of AR-NSCLC PIN and critical AR-NSCLC PIN followed the power law distribution. Their equation are y = 4039.6x ^−1.491^ and y = 394.17x ^−1.062^, respectively. So, they were a scale-free network. Small world networks have a property that mean path length is short [46]. As shown in Fig. 5(C and D), network path length was mostly concentrated in 3–5 steps, which meant that most proteins were closely linked. And AR-NSCLC PIN and critical AR-NSCLC PIN were a small world network.

### Hub genes, module and enrichment analysis

**Fig. 6:**
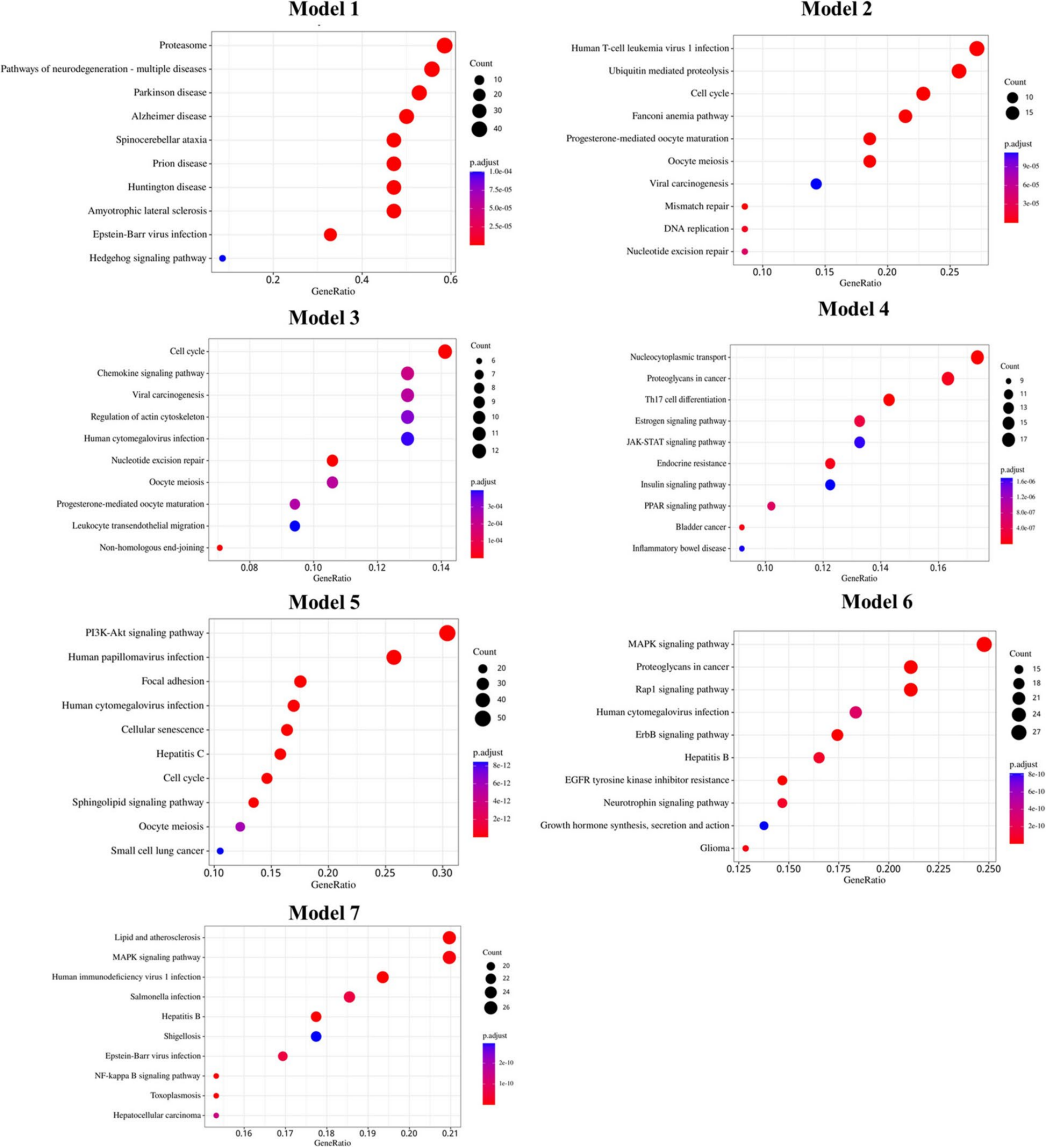
KEGG enrichment analysis of each module. Gene ratio refers to the ratio of enriched genes to all target genes. Counts refer to the number of the enriched genes

After the intersection calculation of the nodes in the top 10 of degree, closeness and betweenness, 6 hub target genes were obtained including TP53, SRC, UBC, CTNNB1, EP300, and RELA. 7 functional modules with a score > 5 were obtained after performing the MCODE (Fig. 4B). KEGG enrichment analysis was performed to discover those pathways enriched by each module target genes. The filter was also set an adjusted* P*-value < 0.05 and *q*‐value < 0.05. The bubble plot of the top 10 KEGG pathways of each module was shown in Fig. 6. KEGG pathways were significantly enriched in JAK-STAT signaling pathway (module 4), PI3K-AKT signaling pathway (module 5), ErbB signaling pathway (module 6), as well as NFkB signaling pathway (module 7).

### Analysis of core genes affecting prognosis in NSCLC patients

**Fig. 7:**
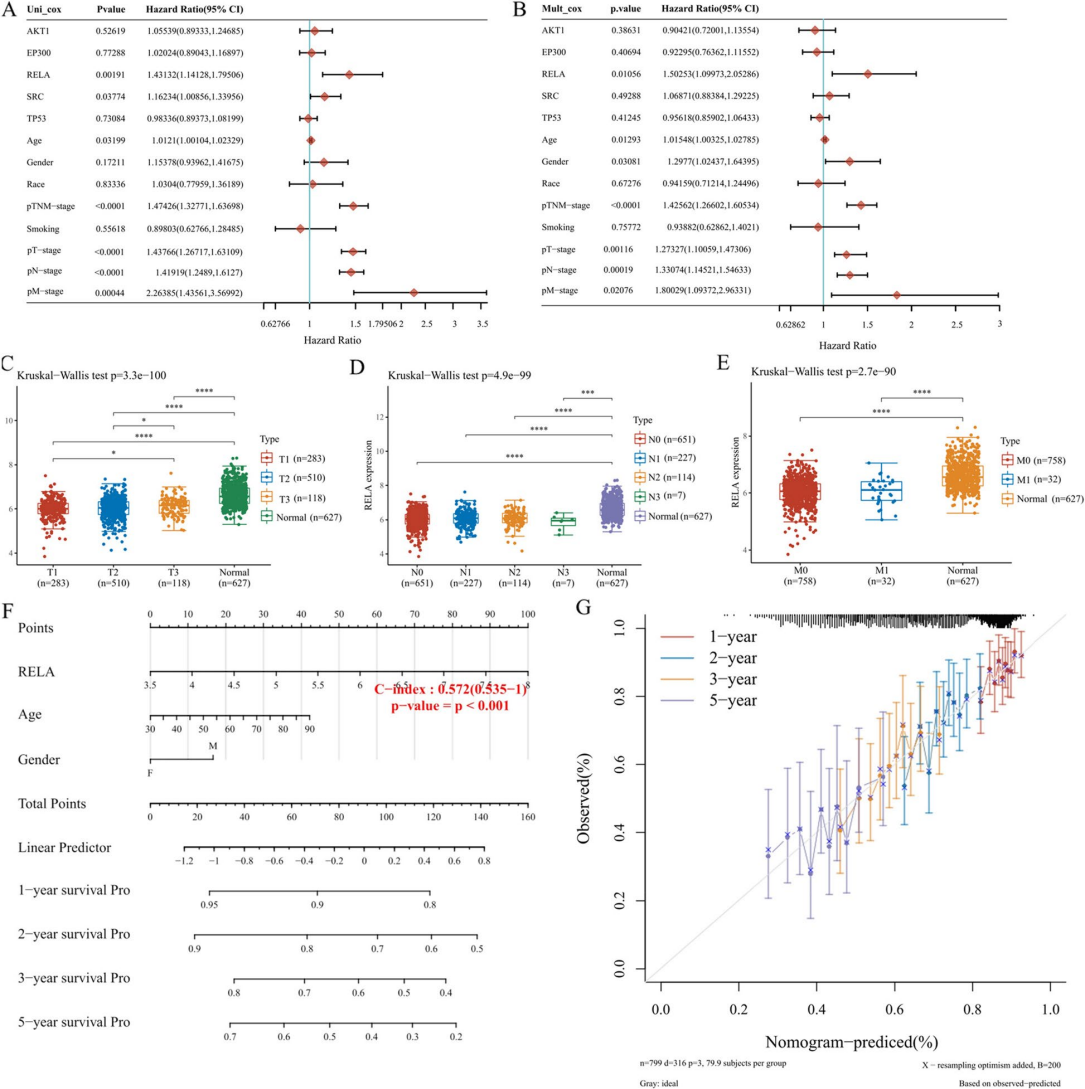
Screening of core genes affecting the prognosis of NSCLC patients. **A** Forest plots based on the results of univariate Cox regression of RELA expression and other clinicopathological factors. **B** Forest plots are based on the results of multivariate Cox regression of RELA expression and other clinicopathological factors. HR and *p*-values were displayed. **C** RELA gene expression distribution in primary tissue and normal tissue of NSCLC tumor tissues. **D** RELA gene expression distribution in lymphoid tissue and normal tissue of NSCLC tumor tissues. **E** RELA gene expression distribution in metastatic grade and normal tissue of NSCLC tumor tissues. *p < 0.05, **p < 0.01, ***p < 0.001 and ****p < 0.0001 by Kruskal–Wallis test. **F** Nomogram predicting the proportion of patients with OS. **G** Plots depict the calibration of model in terms of agreement between predicted and observed OS. Model performance is shown by the plot, relative to the 45-degree line, which represents perfect prediction

Based on TCGA data and clinical information, 6 core genes were used for survival analysis for NSCLC patients. Their relationship with OS was analyzed by univariate and multivariate Cox regression. RELA was associated with OS (*P* < 0.05), which was selected as the prognostic marker for NSCLC (Fig. 7A). The univariate analysis revealed that RELA correlated significantly with a poor OS (hazard ratio)  [34]: 1.50253; 95% confidence interval (CI): 1.09973, 2.05286; *P* = 0.01056). Other clinicopathologic variables including age, pT_stage, pN_stage, and pM_stage associated with poor survival (*P* < 0.05). Gender, race, and smoking did notcorrelate with the prognosis of NSCLC (*P* > 0.05). At multivariate analysis, RELA remained independently associated with OS, with a HR of 1.43132 (CI: 1.14128, 1.79506, *p* = 0.00191), along with age, pT_stage, pN_stage, and pM_stage (Fig. 7B). Thereafter, the difference of RELA expression in NSCLC samples by Kruskal–Wallis test. RELA expression was significantly different in primary tumor size and extent (Fig. 7C), regional lymph nodes (Fig. 7D), and distant metastases (Fig. 7E), further indicating that RELA gene may affect tumor progression and poor prognosis in NSCLC by overexpression. Based on the C-index values, a nomogram integrating the RELA expression, age, pT_stage, pN_stage, and pM_stage was constructed to predict the probability of 1-, 2-, 3- and 5 year OS in NSCLC patients (Fig. 7F). Total points were calculated by adding the points by adding the points of the RELA, age, pT_stage, pN_stage, and pM_stage. The higher the total score, the worse the prognosis of the patient. The calibration plot showed that the nomogram performed well in predicting patient OS according to an ideal model (Fig. 7G).

### Molecular docking analysis

**Fig. 8:**
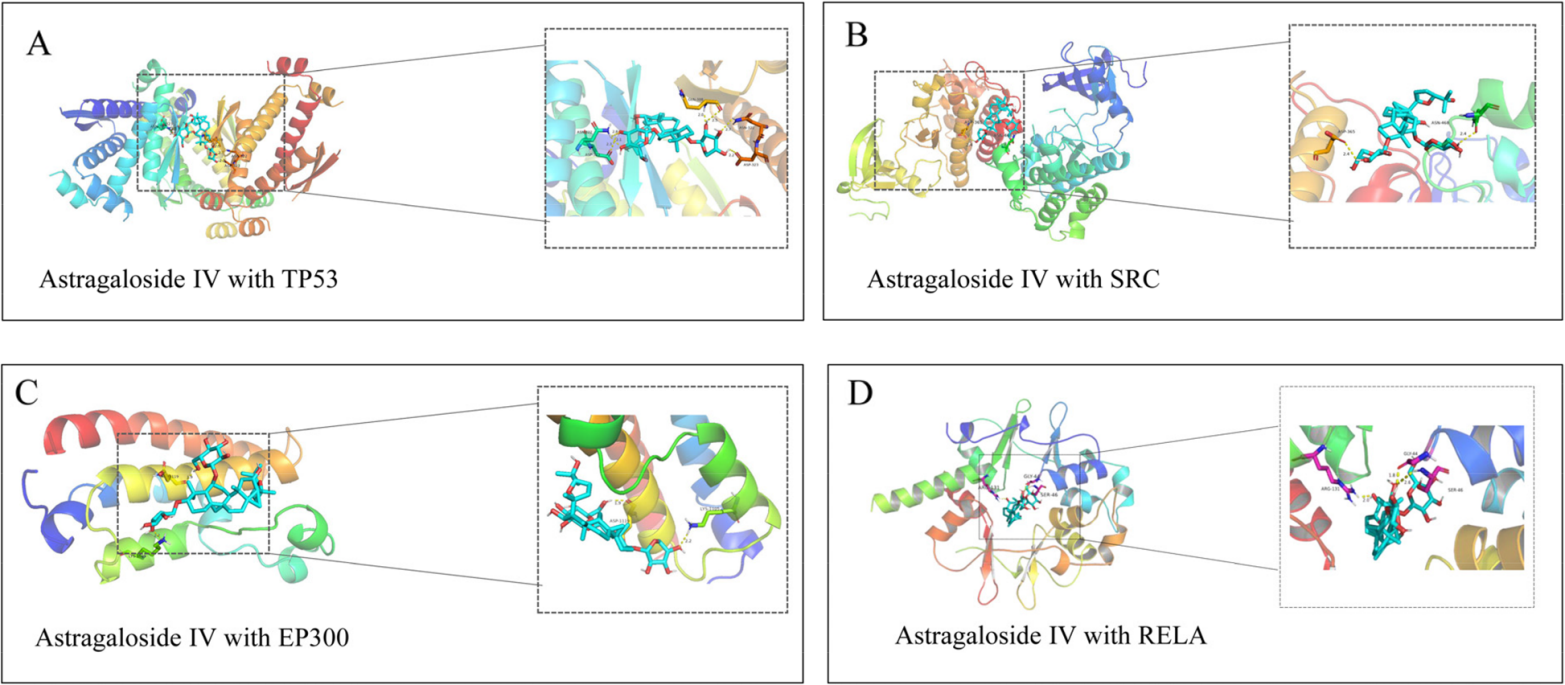
The three-dimensional structure of the interaction between astragaloside IV and hub target genes

**Fig. 9:**
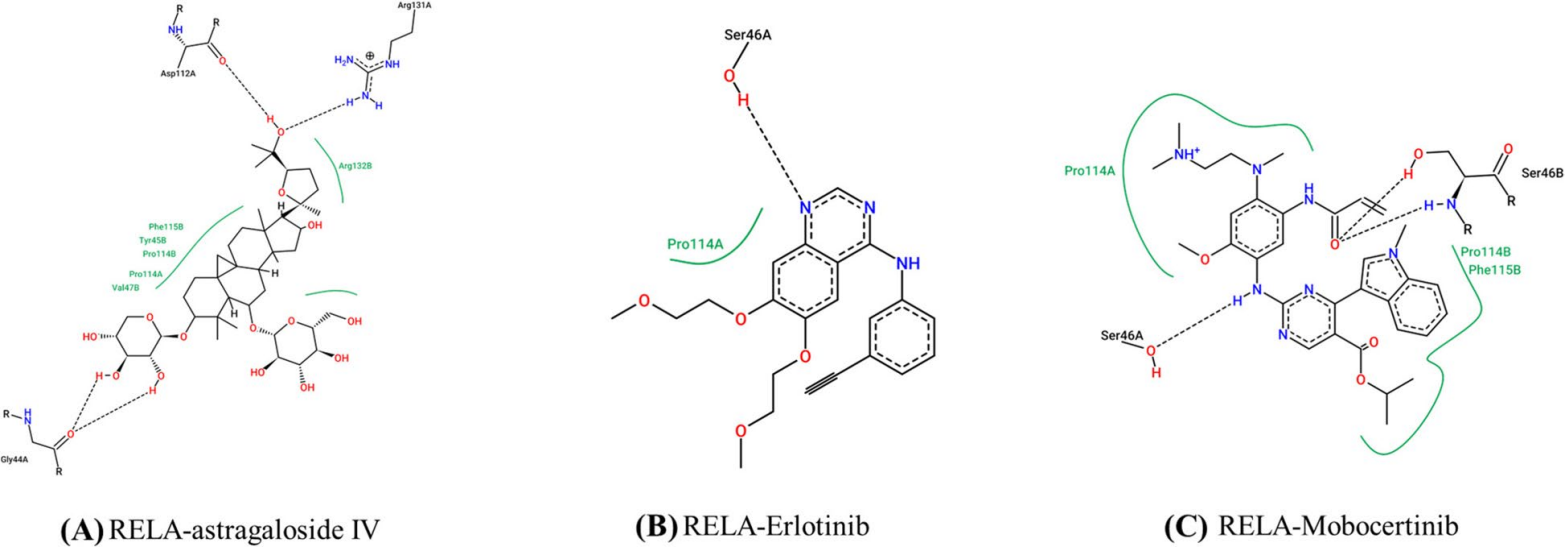
RELA-Ligand interaction diagrams retrieved from Proteins Plus (Pose View). Directed bonds between protein and ligand are drawn as dashed lines and the interacting protein residues and the ligand are visualized as structure diagrams. Hydrophobic contacts are represented more indirectly through spline sections highlighting the hydrophobic parts of the ligand and the label of the contacting residue. **A** RELA-astragaloside IV; **B** RELA-Erlotinib; **C** RELA-Mobocertinib

After the intersection calculation of the hub targets and the proteins participated in the JAK-STAT signaling pathway, PI3K-AKT signaling pathway, ErbB signaling pathway, and NFkB signaling pathway, TP53, SRC, EP300, and RELA were obtained. These proteins were used for molecular docking with astragaloside IV. The components with lower binding energy are generally considered to have higher binding affinity with protein targets. It is generally believed that the binding energy is less than—4.25 kcal·mol^−1^, suggesting that the ligand has a certain binding activity with the receptor, less than—5.0 kcal·mol^−1^ had good binding activity, below -7.0 kcal·mol^−1^ had strong binding activity [47]. According to the molecular docking results, the docking binding energies of the four hub target genes with astragaloside IV were all less than -5 kcal·mol-1, indicating that target genes had good binding activity with astragaloside IV. The binding energy information is shown in Table 3, and the specific binding situation is shown in Fig. 8. Finally, astragaloside IV and Mobocertinib, Erlotinib were interacted with RELA by hydrogen bond. The RELA-ligand interaction diagram is shown in Fig. 9.

**Table 3:** Binding energy between the component and hub target genes

Protein	Binding energy(kcal·mol^−1^)		
	Astragaloside IV	Erlotinib	Mobocertinib
TP53	-8.6	6.5	7.0
SRC	-8.7	-7.8	-7.6
EP300	-7	-6.4	-7
RELA	-8.3	-6.6	-7.7

### Molecular dynamics simulation

**Fig. 10:**
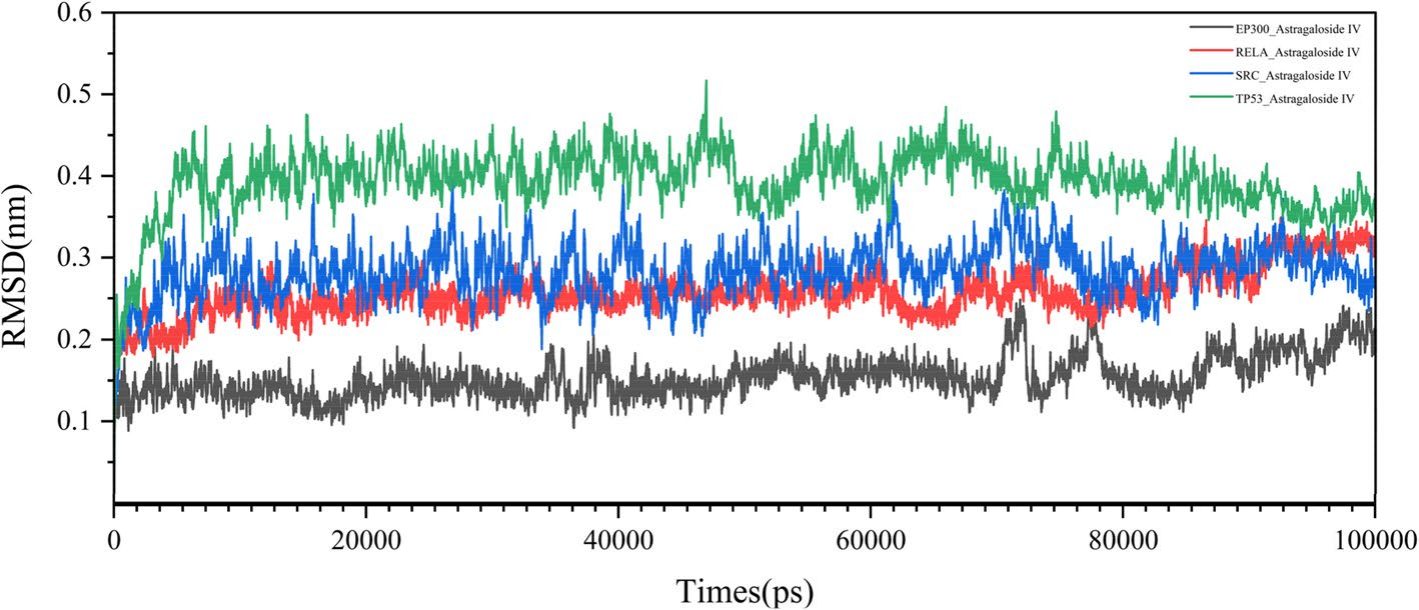
The RSMD simulation results of four complexes

We chose to perform molecular dynamics simulations on the complexes of four proteins (RELA, TP53, EP300, and SRC) bound to Astragaloside IV. The RMSD curve represents positional deviations in the protein. The RELA-Astragaloside IV complex average RMSD value is 0.240771882; the EP300-Astragaloside IV complex average RMSD value is 0.189652057; the SRC-Astragaloside IV complex average RMSD value is 0.283678912; the TP53-Astragaloside IV complex average RMSD value is 0.283678912. As can be seen from Fig. 10, the RELA-Astragaloside IV was in an equilibrium state and tended to balance out the last 10 ns. Meanwhile, the EP300-Astragaloside IV complex had a rise within 80 ns, and tended to balance out the last 10 ns.

In addition, the binding energy of RELA-Astragaloside IV was -37.09 kcal/mol, that of TP53-Astragaloside IV complex was -61.020 kcal/mol, EP300-Astragaloside IV complex was -33.016 kcal/mol, SRC- Astragaloside IV complex was -62.96 kcal/ mol. Molecular dynamics simulations showed that RELA-Astragaloside IV binds tightly to EP300-Astragaloside IV.

## Discussion

**Fig. 11:**
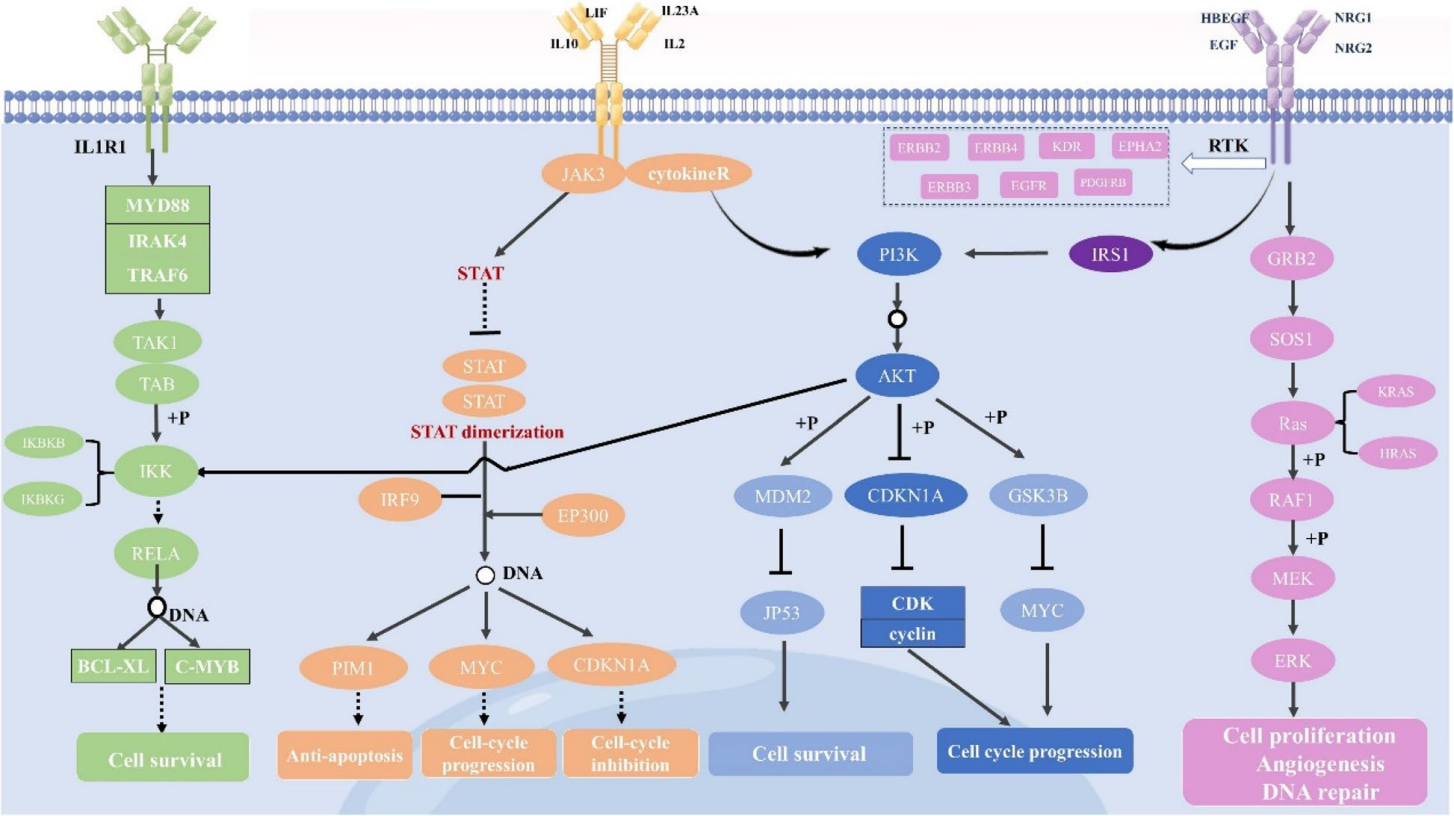
The signaling pathways that regulated by AR on NSCLC

The results of the GO and KEGG enrichment for modules revealed several crucial pathways (signaling pathway JAK/STAT, PI3K-AKT, ERBB and NFκB) that may explain the underlying mechanisms of AR. More interestingly, the four pathways are interrelated (Fig. 11). It is speculated that AR may have the systematically therapeutical effect on NSCLC by multi-pathway including signaling pathway JAK/STAT, PI3K-AKT, ERBB and NFκB.

Module 4 was enriched in JAK-STAT signaling pathway which is closely related to multiple tumorigenesis, progression, invasion, and metastasis [48]. It has been reported that Long noncoding RNA PART1 promotes progression of NSCLC cells via JAK-STAT signaling pathway [49].The genes in this pathway mainly included IL2 (interleukin 2), IL23A (interleukin 23a), LIF (LIF interleukin 6 family cytokine), IL10 (interleukin 10), IL2RA (interleukin 2 receptor subunit alpha), IL2RB (interleukin 2 receptor subunit beta), JAK3 (Janus kinase 3), PIM1 (Moloney murine leukemia virus-1), MYC (MYC proto-oncogene), CDKN1A (cyclin dependent kinase inhibitor 1A).

The JAK/STAT signaling pathway promotes the cytokine-mediated cell activation in a simple and efficient way [50]. Binding of the cytokines to their receptors activates janus kinases(JAKs) [51]. The activated JAK phosphorylates the receptor, activates and phosphorylates its main substrate STAT. Phosphorylated STAT dimerizes with other members of STAT family with conserved SH2 domains. The dimer is then translocated into the nucleus and binds to the specific adjustment zones of DNA sequences to activate PIM1, MYC and CDKN1A [52], leading to anti-apoptosis and cell cycle inhibition. In Richeng Jiang’s observation, nuclear overexpression of PIM1 correlated with LN metastasis, histology and poor survival in NSCLC [53]. The targeting of Myc in lung cancer is potentially an unprecedented opportunity for inhibiting a key player in tumor progression and maintenance and therapeutic resistance [54]. And its aberrant overexpression has been reported in about 30–75% of NSCLC cases [55]. Besides, phosphorylated JAK allows for PI3K activation, which in turn activates the downstream cascade of the PIK3/AKT signaling pathway [56].

Module 5 was related to the PI3K-AKT signaling pathway mainly including PIK3CA, PIK3R1, EGFR(Epidermal growth factor receptor), ERBB2 (epidermal growth factor receptor 2), ERBB3(epidermal growth factor receptor 3), KDR(VEGF receptor 2), EPHA2(Ephrin type-A receptor 2), PDGFRB(platelet-derived growth factor receptor beta), IRS1(insulin receptor substrate 1), CDK2(cyclin-dependent kinase 2), CDK4(cyclin-dependent kinase 4), CDK6(cyclin-dependent kinase 6), GSK3B(glycogen synthase kinase 3 beta), SOS1(Son of sevenless 1), HRAS(HRas proto-oncogene), KRAS(Kirsten rat sarcoma viral oncogene).

In NSCLC, the PI3K-Akt pathway has been heavily implicated in both tumorigenesis and the disease progression [57]. PIK3CA and PIK3R1 belong to class IA PI3K that are typically activated by tyrosine receptor kinases (RTKs) such as KDR, EPHA2, PDGFRB, EGFR, ERBB2, ERBB3 and ERBB4 [58]. EGFR has become an important therapeutic target for the treatment of NSCLC. Inhibitors that target the kinase domain of EGFR have been developed and are clinically active. The presence of PIK3CA gene mutations or amplifications has been found in a diverse range of malignancies [59]. PI3K phosphorylates phosphatidylinositol 4,5‐bisphosphate (PIP2), to produce phosphatidylinositol (3,4,5)–trisphosphate (PIP3 [60]). Then, PIP3 activated Akt directly via phosphorylation [61]. Akt consists of three homologues, Akt1, Akt2 and Akt3 [62]. Akt activation subsequently leads to a number of potential downstream effects. It can result in inhibition of cyclin-dependent kinase (CDK), RAF1 and also GSK3B [63], leading to regulation of cell cycle progression, cell survival and cell proliferation. The Phosphorylate Akt may also active MDM2, which causes downregulation of p53‐mediated apoptosis and forkhead transcription factors that produce cell‐death promoting proteins [58]. The nuclear factor kappa‐light‐chain‐enhancer of activated B cells (NFκB) transcription factor plays a crucial role in the consequences of PI3K/Akt pathway activation. NFκB regulates gene expression of hundreds of genes which are implicated in apoptosis, cell cycle control, immune modulation, cell survival and cell adhesion and differentiation [64]. Akt prevents negative regulation of NFκB by the IκB family, and in particular IκBα. IκBα removes NFκB from DNA and returns it to the cytoplasm [65].

Module 6 was closely related to ErbB signaling pathway. It included EGF (epidermal growth factor), HBEGF (heparin binding EGF like growth factor), NRG1 (neuregulin-1), NRG2 (neuregulin-2), ERBB4 (epidermal growth factor receptor 4), SRC(proto-oncogene tyrosine-protein kinase Src), RAF1(Raf-1 proto-oncogene, serine/threonine kinase).

The ErbB signaling pathway performs important role in the molecular pathogenesis of lung cancer [66]. The pathway was extensively analyzed and found to be key to the unremitting growth and development of carcinoma cells. The ligands (EFG, HBEGF, NRG1 and NRG2) actived the ErbB receptors though the direct or indirect way which included EGFR and ERBB4. Homo- or heterodimerization of these receptors occurs after ligand binding, leads to activation of downstream signaling cascades like SRC, PI3K/AKT and Ras/Raf/MEK/ERK. In ERK cascade, the SHC and GRB2 phosphotyrosine-binding adaptors link phosphorylated receptors, through a guanine nucleotide exchange protein (SOS) and a small GTP-binding protein (RAS), to a linear cascade culminating in ERK1 and ERK2, which translocate to the nucleus to stimulate various transcription factors [67]. NRG1 has been revealed fusions to exist at low frequencies across multiple tumor types, though the largest number of cases have been identified in NSCLC [68]. SRC are frequently expressed and activated in lung cancer which involved in the proliferation, survival, angiogenesis, invasion, and migration of various types of cancer cells [69].

Module 7 was associated with the NFKB signaling pathway which is considered the classical, or canonical pathway of NFkB [70]. The proteins included IL1R1(interleukin-1 receptor 1), MYD88(MYD88 innate immune signal transduction adaptor), TRAF6(TNF receptor associated factor 6), IRAK4(interleukin 1 receptor associated kinase 4), IKBKB (inhibitor of nuclear factor kappa B kinase subunit beta), IKBKG(inhibitor of nuclear factor kappa B kinase regulatory subunit gamma), BCL2L1(BCL2 like 1), BCL2(BCL2 apoptosis regulator), BIRC2(baculoviral IAP repeat containing 2).

In this canonical NFkB signaling pathway, the activation of IL1R1 phosphorylated IKBKB in the IKK complex consisting two catalytic subunits (IKBKA and IKBKB) and a regulatory subunit (IKBKG), and this process is mediated by the adapter protein MYD88. Then, IKK phosphorylates IkB, thus tagging it for proteasomal degradation and freeing the NFkB subunit dimers (p50/p65). NFkB dimers translocate to the nucleus where they bind to specific DNA sequences in the promoter region of a wide array of genes [71]. Meta-analysis concluded that NFκB expression may be a potential unfavorable prognostic marker for NSCLC patients [72].

Based on survival analysis, RELA could influence the survival of NSCLC patients. And there was a positive correlation between RELA expression and tumor grade, including pT_stage, pN_stage, and pM_stage. It has prognostic value for patients and can be used to diagnose diseases or indicate the severity of diseases. The RELA played a central role in the NFκB pathway [73]. Lin HC’s research showed that RELA attenuation significantly inhibited the proliferation and induced apoptosis of NSCLC cells in vitro, suggesting the indispensable role it has in lung cancer [74]. More interestingly, AR can inhibit the RELA expression according to western bolting test. RELA might be of greater value to be used for diagnosis, treatment and prognosis of NSCLC patients.

**Fig. 12:**
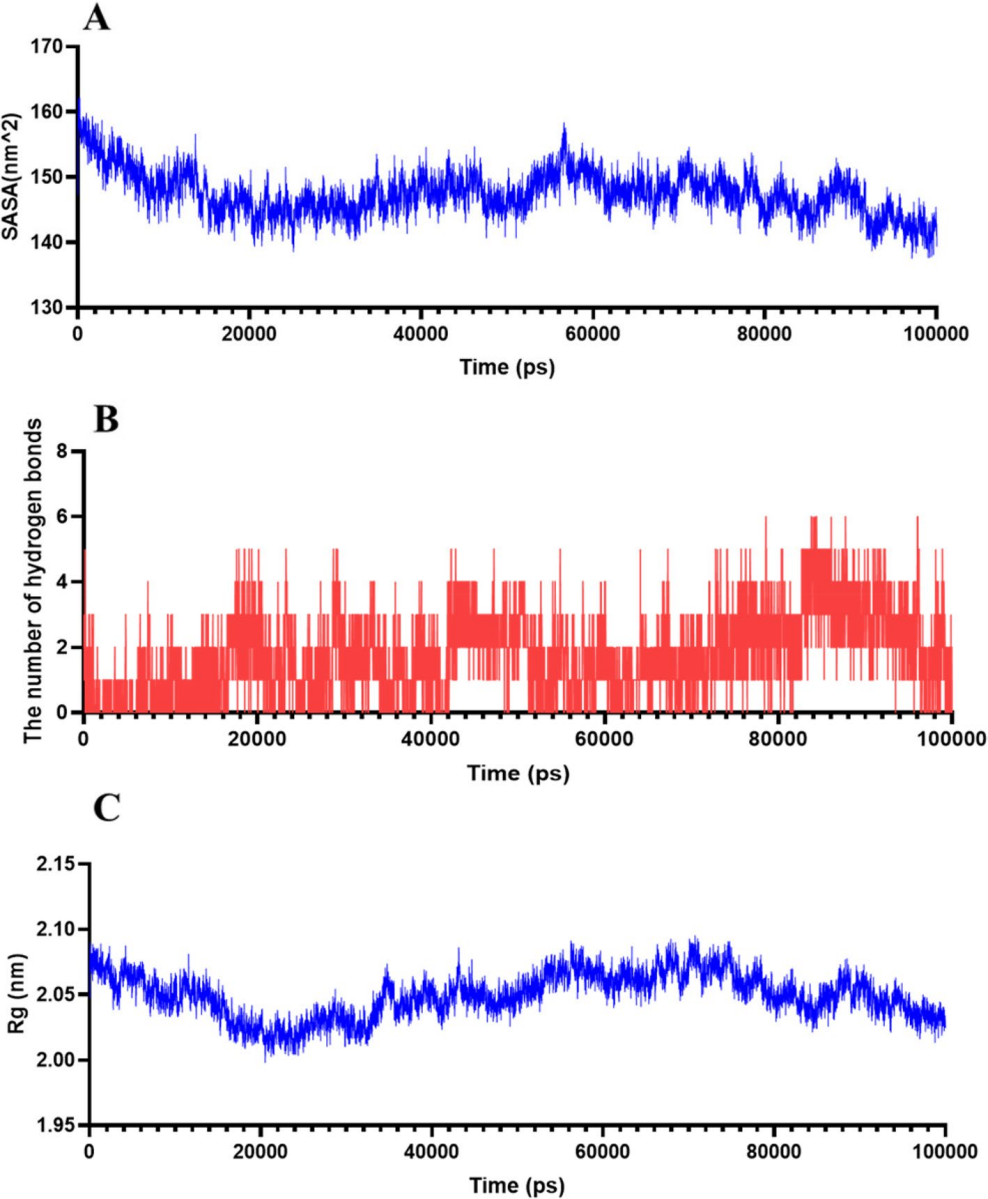
Simulation analysis of molecular dynamics of RELA-Astragaloside IV complexes. **A** The SASA between the RELA and astragaloside IV; **B** The HBond between astragaloside IV and RELA; **C** The Gyrate between astragaloside IV and RELA proteins

Combined with molecular simulations of RSMD, RSMF, and binding free energy data, we fouce that the RELA binds better to Astragaloside IV, and thus we performed an in-depth analysis of the RELA- Astragaloside IV complex with multiple metrics. The solvent accessible surface area (SASA) is a measure of a protein’s surface area. We determined SASA between the RELA and astragaloside IV (Fig. 12A). The findings demonstrated that the RELA’s SASA value was high prior to its binding to the small molecule but decreased following its binding, indicating that astragaloside IV and RELA binding reduced the protein’s surface area. Hydrogen bonding (HBond) of RELA and astragaloside IV interaction was assessed by the HBond (Fig. 12B). The results showed that many hydrogen bonds were formed between astragaloside IV and RELA, mainly between some key residues in the protein and some important groups in the small molecule. The radius of Gyration (Gyrate) is an indicator of the overall compactness of the protein, the Gyrate between astragaloside IV and RELA showed that the RELA value of proteins combined with small molecules decreased compared with that of individual protein molecules, indicating that the binding of small molecules makes the protein molecules more compact (Fig. 12C).

**Fig. 13:**
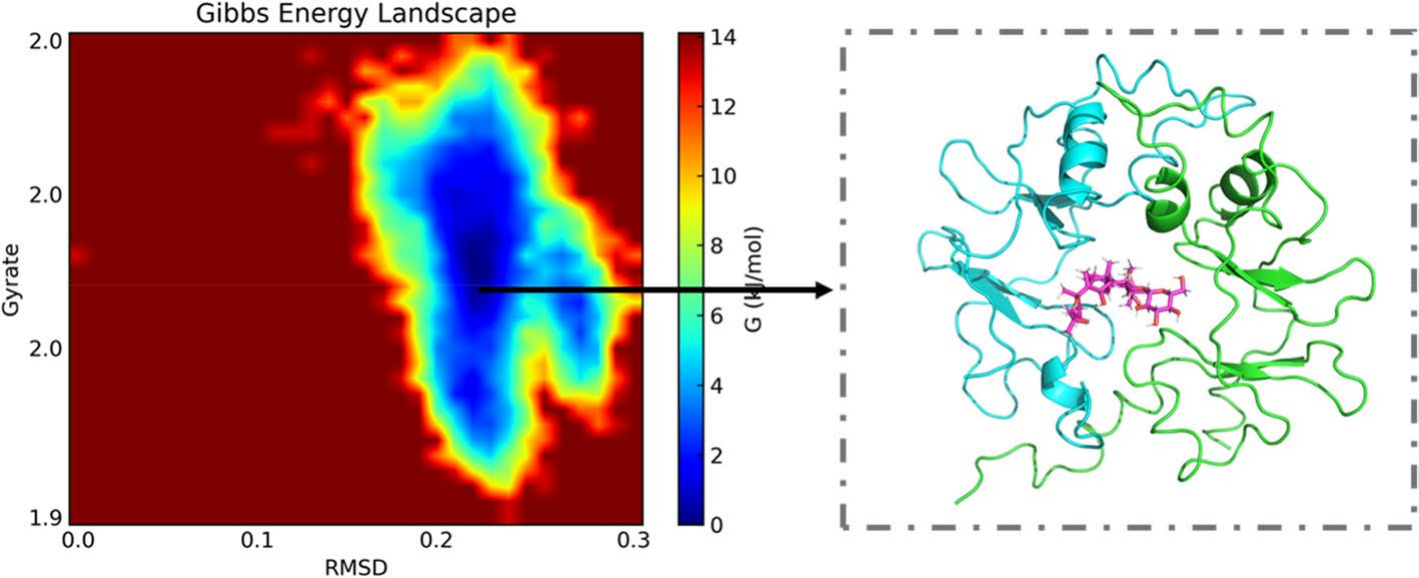
Free energy morphology of RELA-Astragaloside IV complex

The free energy landscape (FEL) of the complex of RELA -Astragaloside IV was created by combining two indicators, RMSD and R(g), as shown in Fig. 13. During the simulation process, there are two low-energy regions on the free-energy maps, indicating that there are two relatively stable states in the molecular structure, which are mainly concentrated around 30 ns and 80 ns.

## Conclusion

Pharmacological mechanisms of AR on NSCLC were explored, promoting the clinical application of AR in treating NSCLC. UPLC-Q-Orbitrap HRMS analysis of AR which provided chemical component information references for further researches. Based on network pharmacology approach, TP53, SRC, UBC, CTNNB1, EP300, and RELA were identified as hub genes. KEGG results showed that AR may produce curative effects on signaling pathway JAK-STAT, PI3K-AKT, ErbB, and NFkB. Of these hub genes, TP53, SRC, EP300, and RELA participated in the above signaling pathways and had great docking energy with the key components of AR. The survival analysis revealed that RELA was proved to have prognostic value on NSCLC patients’ survival. These findings provided references for further researches.

### Supplementary Information

**Additional file 1: Supplementary Table 1.** Pubchem compound information of drugs.

## Data Availability

All data generated or analyzed during this study are included in this published article.
