# Supplementary material for: Network module analysis and molecular docking-based study on the mechanism of astragali radix against non-small cell lung cancer
Source: BMC Complement Med Ther. 2023 Sep 28;23:345. doi: 10.1186/s12906-023-04148-9 (PMC10537544; doi:10.1186/s12906-023-04148-9)
Supplement: Supplementary file 1 — Additional file 1: Supplementary Table 1. Pubchem compound information of drugs. [file 12906_2023_4148_MOESM1_ESM.docx]

Supplementary Table 1 Pubchem compound information of drugs.

| Properties | Mobocertinib | Erlotinib | Astragaloside IV |
| --- | --- | --- | --- |
| Compound ID | CID 118607832 | CID 176870 | CID 13943297 |
| Mol.Wt (g/mol) | 585.7 | 393.4 | 785.0 |
| Mol.Formula | C_32_H_39_N_7_O_4_ | C_22_H_23_N_3_O_4_ | C_41_H_68_O_14_ |
| XLogP3-AA | 4.4 | 3.31 | 1.26 |
| H-Bond Donor | 2 | 1 | 9 |
| H-Bond Acceptor | 9 | 7 | 14 |
| Rotatable Bond | 13 | 11 | 7 |
| Exact Mass | 585.30635275 | 393.16885622 | 784.46090684 |
| TPSA | 113.9 | 74.7 | 228.2 |
| Heavy Atom | 43 | 29 | 55 |
| Complexity | 935 | 525 | 1460 |
| IUPAC Name | propan-2-yl 2-[4-[2-(dimethylamino)ethyl-methylamino]-2-methoxy-5-(prop-2-enoylamino)anilino]-4-(1-methylindol-3-yl)pyrimidine-5-carboxylate | N-(3-ethynylphenyl)-6,7-bis(2-methoxyethoxy)quinazolin-4-amine | (2R,3R,4S,5S,6R)-2-[[(1S,3R,6S,8R,9S,11S,12S,14S,15R,16R)-14-hydroxy-15-[(2R,5S)-5-(2-hydroxypropan-2-yl)-2-methyloxolan-2-yl]-7,7,12,16-tetramethyl-6-[(2S,3R,4S,5R)-3,4,5-trihydroxyoxan-2-yl]oxy-9-pentacyclo[9.7.0.01,3.03,8.012,16]octadecanyl]oxy]-6-(hydroxymethyl)oxane-3,4,5-triol |
| Canonical SMILES | CC(C)OC(=O)C1=CN=C(N=C1C2=CN(C3=CC=CC=C32)C)NC4=C(C=C(C(=C4)NC(=O)C=C)N(C)CCN(C)C)OC | COCCOC1=C(C=C2C(=C1)C(=NC=N2)NC3=CC=CC(=C3)C#C)OCCOC | CC1(C(CCC23C1C(CC4C2(C3)CCC5(C4(CC(C5C6(CCC(O6)C(C)(C)O)C)O)C)C)OC7C(C(C(C(O7)CO)O)O)O)OC8C(C(C(CO8)O)O)O)C |
